# The Beat

**DOI:** 10.1289/ehp.120-a270b

**Published:** 2012-07-02

**Authors:** Erin E. Dooley

## Study Examines Epigenetic Effects of Fungicide Exposure

A mother’s environmental exposure can result in transgenerational effects that are inherited through successive generations, even in the absence of exposure of the offspring. A new proof-of-principle study reports that great-grandchildren of rats exposed to the common fungicide vinclozolin showed a more profound behavioral and neurodevelopmental response to stress, compared with offspring of unexposed rats.^1^ Following exposure to stressful situations during adolescence, ancestrally exposed rats were heavier, more anxious, and less sociable than unexposed offspring.

## Government Announces Action Plan to Address Asthma Disparities

Asthma affects an estimated 7 million U.S. children. Asthma rates among black and Hispanic children are more than double the rate among white children; poverty also is associated with disproportionately higher rates. In May 2012 a partnership of federal government agencies launched the Coordinated Federal Action Plan to Reduce Racial and Ethnic Asthma Disparities. The action plan will coordinate efforts in four areas: reducing barriers to asthma care, building capacity for community-based asthma care, targeting services to those most affected by asthma, and increasing efforts to understand the causes of asthma and validate interventions to prevent the onset of asthma.^2^

**Figure:**
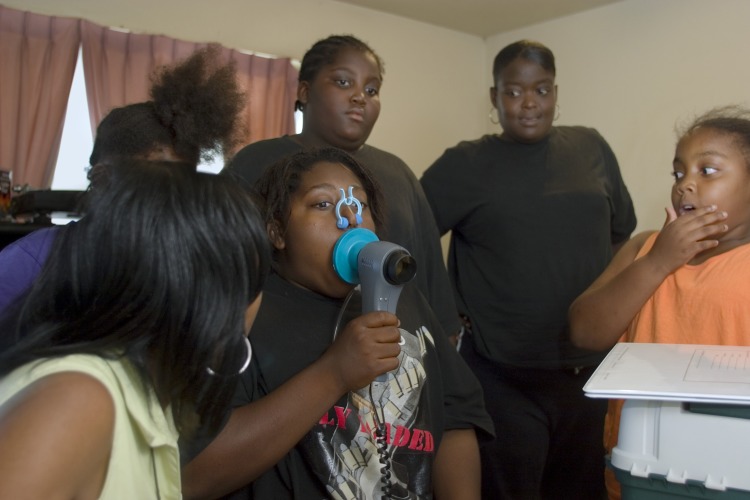
Children at a Baltimore housing project test their breathing ability using a peak flow monitor. © Bob Sacha/Corbis

## Pollen Allergenicity Is Higher along High-Traffic Roads

When researchers conducted protein immunoblot analyses of ragweed pollen samples from different areas, they found on average that samples collected alongside highly traveled roadways exhibited a statistically higher IgE-binding signal—a measure of allergenicity—than samples from less-polluted vegetated areas. Pollen from high-traffic areas also expressed a greater variety of allergens. Although the researchers couldn’t determine whether specific environmental factors induced the higher reactivity of pollen in plants from the more polluted sampling areas, they did confirm the plants in these areas produced more reactive pollen allergens.^3^

## A Clearer Picture of Graphene’s Properties

Graphene nanoplatelets are being incorporated into applications such as electronics, semiconductors, batteries, and industrial coatings. A new study reports that their unusual dimensions (10 nm thick by 5 µm in diameter) make graphene nanoplatelets small enough to deposit deep into the lungs but too large for the immune system to remove them.^4^ The authors say this raises the potential for pleural inflammation and disease over time. They propose that occupational risks from graphene platelet exposure could be lessened if manufacturers produce smaller platelets, which could be cleared by macrophages.

**Figure:**
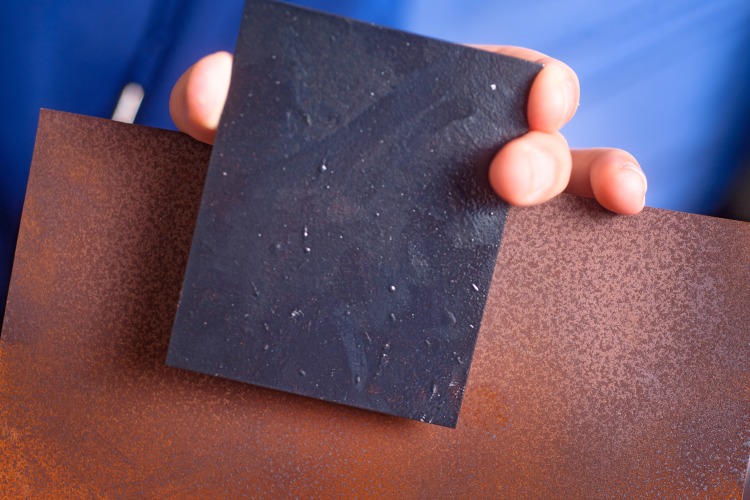
A graphene-based coating under development at the University at Buffalo keeps steel rust-free, potentially replacing coatings that contain toxic hexavalent chromium. © 2012 University at Buffalo/Sarbajit Banerjee

## Alaskan Law Allows Use of Gravel Containing Asbestos

Alaska is home to large deposits of ore peppered with naturally occurring asbestos. Until now, the discovery of such asbestos in the course of construction projects has been enough to halt work. In an effort to restart stalled development, Alaskan governor Sean Parnell passed a law allowing construction projects to use local gravel containing naturally occurring asbestos.^5^ If the gravel being used contains more than 0.25% asbestos, the builders must register with the state and provide a site-specific plan for meeting asbestos safety standards. The new law also absolves construction companies of responsibility in lawsuits that may arise from health effects resulting from asbestos exposure. A body of scientific evidence supports the position that there is no safe level of exposure to asbestos.^6^

**Figure:**
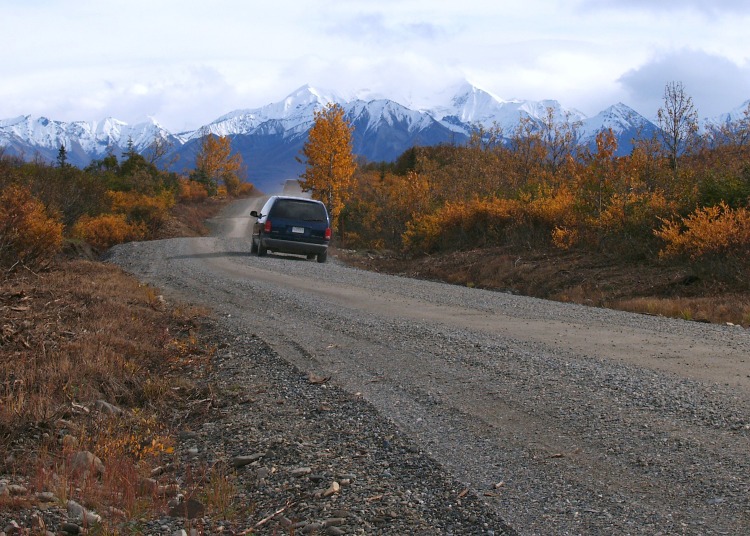
Alaskan construction may get a boost from a new law that allows the mining and use of asbestos-containing gravel. © Laura Lohrman Moore/Shutterstock.com
